# Sleep disorders mediate the link between childhood trauma and depression severity in children and adolescents with depression

**DOI:** 10.3389/fpsyt.2022.993284

**Published:** 2022-10-28

**Authors:** Bei Luo, Yingying Yang, Dapeng Zhang, Qing Zhang, Zhichun Liu, Song Wang, Yudong Shi, Lei Xia, Jiawei Wang, Zhiwei Liu, Feng Geng, Changhao Chen, Xiangwang Wen, Xiangfen Luo, Kai Zhang, Huanzhong Liu

**Affiliations:** ^1^School of Mental Health and Psychological Sciences, Anhui Medical University, Hefei, China; ^2^Department of Psychiatry, Chaohu Hospital of Anhui Medical University, Hefei, China; ^3^Anhui Psychiatric Center, Hefei, China; ^4^Department of Psychiatry, Fuyang Third People's Hospital, Fuyang, China; ^5^Department of Psychiatry, Bozhou People's Hospital, Bozhou, China; ^6^Department of Psychiatry, Hefei Fourth People's Hospital, Hefei, China; ^7^Department of Psychiatry, Suzhou Second People's Hospital, Suzhou, China; ^8^Department of Psychiatry, Ma'anshan Fourth People's Hospital, Maanshan, China; ^9^Department of Psychiatry, The Second Affiliated Hospital of Bengbu Medical College, Bengbu, China

**Keywords:** insomnia, childhood trauma, sleep disorders, children and adolescence, depression

## Abstract

**Background:**

Childhood trauma is closely related to the onset of depression and more severe depressive symptoms; however, the specific mechanisms are unclear. We aimed to examine the relationship between childhood trauma and sleep disorders in children and adolescents with depression and to explore further the role of sleep disorders in the relationship between childhood trauma and depression severity.

**Methods:**

A total of 285 children and adolescents with depression completed all scale assessments, including the Childhood Trauma Questionnaire, Self-Reported Insomnia Severity Index and Epworth Sleepiness Scale, and the Center for Epidemiologic Studies Depression Scale. A simple mediation model was used as a theoretical model to examine whether sleep disorders could mediate the relationship between childhood trauma and depression severity.

**Results:**

Among children and adolescents with depression, childhood trauma is about 78.9%. Compared with patients without childhood trauma, patients with childhood trauma had a higher incidence of sleep disorders (*Z* = 17.59, *P* < 0.001), which were characterized by insomnia (*Z* = 14.45, *P* < 0.001), not hypersomnia (*Z* = 2.77, *P* = 0.096). Different childhood trauma subtypes significantly affected sleep disorders and insomnia (all *P* < 0.05). Insomnia partially mediated the relationship between childhood trauma and depression severity, and the mediating effect accounted for 35.90%.

**Conclusion:**

This study found a high rate of concurrent childhood trauma and insomnia among children and adolescents with depression. Insomnia, as a mediator between childhood trauma and depression severity, partially mediates the relationship.

## Introduction

There is a high incidence of mental disorders in children and adolescents, of which depression is the most common type (1). Studies have shown that depression is the leading cause of disability and death in adolescents (2). The prevalence of depression among adolescents and young adults has increased in recent years (3, 4). In 2021, a national epidemiological survey of mental illness among Chinese children and adolescents found that the prevalence of depression is about 3.0% (5). Longitudinal studies found that more than half of people who experienced depression during adolescence relapsed at least once in adulthood (6, 7). In addition, depression in children and adolescents has serious negative consequences, such as poor relationships with peers and families, poor academic performance, addiction to the Internet, and other bad behavior. Depression in children and adolescents often indicates chronic or recurrent disorders as well as a wide range of psychosocial difficulties and health problems (8).

The pathogenesis of depression is a complex process influenced by many factors, including genetic and environmental factors (9–12). Among environmental factors, there was substantial evidence that childhood trauma plays a vital role in the pathogenesis of depression, and adolescents who have experienced childhood trauma may be more likely to develop depression than those not (13, 14). A growing body of epidemiological and clinical studies have linked childhood trauma to lifelong depression (15–17). Research has shown that childhood trauma is related to the severity and chronic course of depressive symptoms (18, 19). In addition, different subtypes of childhood trauma have been shown to have varying degrees of impact on depression (20–22).

How childhood trauma affects depressive symptoms and the mechanism of these neurobiological changes is unclear. It may lead to long-term chronic neurobiological changes that predispose individuals to depression. Most current research focuses on the stress response system and speculates the development of depression with the changes in the stress response system (9, 10, 23). A significant correlation between stress-related epigenetic changes and depression has been confirmed (24). Exposure to high-stress levels in childhood may lead to excessive or insensitive neuroendocrine stress responses in adulthood, leading to depression (17). This phenomenon is also observed in adolescents (25). Sleep disorders and circadian rhythm disorders, closely associated with the stress response system, may be the underlying pathophysiological mechanisms between childhood trauma and depression (23, 26).

Sleep disorders are common among adolescents, with insomnia and hypersomnia particularly prominent (27). Several studies have linked childhood trauma exposure to post-exposure insomnia, nightmare-related distress, and types of sleep disorders (28, 29). Sleep disorders, such as subjectively experienced difficulty falling asleep, easy awakening, and shortened sleep time, are common symptoms after trauma exposure in the short and long term (30, 31). The effects of adverse childhood experiences on sleep are also observed in adolescents (32). In 2016, a large sample of adolescent-based studies found a dose–response relationship between the number of childhood adversity reports and the risk of insomnia (33).

Furthermore, as in adults, there is a reciprocal relationship between sleep disorders and depression in adolescents, with a high incidence of sleep disorders in depressed adolescents (34), and sleep disorders were significantly correlated with the severity of depression (35), with insomnia being the most common sleep disorders. Insomnia is a strong predictor of depression (36). Previous studies have also found that depressed patients with hypersomnia have more severe depressive symptoms (37). Sleep problems after experiencing traumatic events can increase the risk of mental health problems associated with trauma (38). Based on the above, we hypothesize that sleep disorders may be an underlying mechanism for developing depression severity from childhood trauma.

Although childhood trauma and sleep disorders are common among children and adolescents with depression, previous studies have not examined the common link between depression severity and the two in this population. Only in 2020, a study of patients with schizophrenia and bipolar disorder found that childhood trauma and insomnia symptoms coexist, with insomnia partially moderating the relationship between childhood trauma to dysfunction and the severity of clinical symptoms (39). Therefore, this study investigated the relationship between childhood trauma and sleep disorders in children and adolescents with depression. In addition, we created a mediation model to examine further whether the relationship between childhood trauma to depression severity is mediated by sleep disorders.

## Methods

### Participants

From January to July 2021, based on the inclusion and exclusion criteria, children and adolescent depression patients were consecutively recruited in outpatient clinics and wards of various centers of seven hospitals in Anhui, China, including general tertiary hospitals and specialized tertiary hospitals. Clinicians used the Diagnostic and Statistical Manual of Mental Disorders, Fifth Edition (DSM-5) to diagnose all patients with depression. The inclusion criteria were as follows: (1) meet the diagnostic criteria of depression in DSM-5; (2) age 18 years and below; (3) obtain written informed consent from subjects or legal guardians. Participants will be excluded when the following conditions exist: (1) severe neurological diseases; (2) intellectual disability of patients; (3) infectious diseases and immune system diseases; (4) patients with severe physical diseases; (5) unable to cooperate with the assessment or non-cooperating. Our study was approved by the ethics committees of Chaohu Hospital of Anhui Medical University and various centers, and the ethics number is 202009-kyxm-04. All subjects participated in the study voluntarily, and the informed consent was signed by the subjects or their guardians.

### General information and clinical features

Using the self-designed general questionnaire, the gender, age, BMI, grade (junior high school, senior high school, or above), sibling, physical illness, parental marriage (normal marriage, divorce /remarriage, separation, etc.), and family relationships (good, general, and bad) of all participants were collected. BMI's formula is weight (kg)/height (m)^2^, in which height and weight are measured when participants take off their shoes and wear light clothes.

### Childhood trauma

The Childhood Trauma Questionnaire Short Form (CTQ-SF) has been reported to have acceptable consistency and test–retest reliability (40). The Chinese version of the CTQ-SF has good validity and reliability for adolescents (41, 42). This scale contains 28 items, each of which uses a quintile scale ranging from 1 (never), 2 (occasionally), 3 (sometimes), and 4 (often) to 5 (always) to describe the response to each item. This study used its five subscales besides the total childhood trauma score: physical abuse, emotional abuse, sexual abuse, emotional neglect, and physical neglect.

Childhood trauma data were used as dichotomous and continuous variables. The dichotomy was to classify childhood trauma as “no childhood trauma” (no or mild trauma exposure) and “childhood trauma” (moderate or severe exposure). When emotional abuse ≥ 13, physical abuse ≥ 10, sexual abuse ≥ 8, emotional neglect ≥ 15, and physical neglect ≥ 10 in the subscale, as long as one subscale score meets the above conditions for moderate and severe trauma exposure, this study is regarded as “childhood trauma” (43).

### Sleep disorders

Sleep disorders include insomnia, hypersomnia, disturbed sleep–wake rhythms, and abnormal activities and behaviors during sleep. Here, we focus on the two most common forms of sleep disorders—insomnia and hypersomnia. We defined the absence of both insomnia and hypersomnia as having no sleep disorders; otherwise, it was defined as sleep disorders.

#### Insomnia severity index

ISI was used to assess the nature and impact of insomnia in the last month and to measure the self-perceived severity of insomnia symptoms (44). It is a seven-item self-report questionnaire with a total score of 0–28. ISI has been verified in adolescents in Hong Kong, China, and has been shown to have good psychometric properties (45). It has been determined that the best cutoff value for monitoring adolescent clinical insomnia is nine points (45). We define <9 points as no clinical insomnia and ≥9 points as clinical insomnia. In this study, it is applied as a continuous variable and a binary variable.

#### Epworth sleepiness scale for children

We used the ESS-CHAD to assess sleepiness symptoms (46), a reliable and internally valid measure of daytime sleepiness in adolescents (47, 48). The scale has a total of eight items, with a total score of 0–24. We defined ESS as ≤ 10 as no hypersomnia and >10 as hypersomnia.

### The center for epidemiologic studies depression

This scale is widely used to assess depression severity at various ages and has been reported to have good validity and reliability in adolescents (49, 50). The scale consists of 20 items with a score of 0–60. The higher the scores, the more severe depression. In this study, we analyzed the CES-D scale data as continuous variables.

### Statistical analysis

We use SPSS23.0 software to analyze the data. First, the depression patients with or without childhood trauma were compared. We appropriately used the independent samples *t*-test and Mann–Whitney *U*-test for continuous variables. The chi-square test was used for categorical variables. Second, the Mann–Whitney *U*-test was used to analyze differences in childhood trauma total scores and subtypes (continuous variables) and sleep disorders (insomnia, hypersomnia, dichotomous variables). The ultimate goal of this study was to examine whether sleep disorders mediate between childhood trauma and depression severity. To achieve this, we created the mediation model, where childhood trauma was selected as the independent variable, sleep disorders were selected as mediating variables, and depression severity was selected as the dependent variable. The relationship between childhood trauma, sleep disorder, and depression severity has long been established, and our model is clinically logical and supported by the corresponding literature (23, 25, 26, 35, 38). We then performed a series of tests on the model, using Spearman's correlation analysis to calculate independent variables (total childhood trauma score, continuous variable), mediator variables (different sleep disorders; continuous variable), and correlations between severity of depression. Finally, we performed a mediation analysis based on regression analysis using SPSS, applying the simple mediation model, using 5,000 bootstrapped samples. The total childhood trauma score was set as the independent variable, sleep disorders (insomnia; hypersomnia) as the mediator variable, and depression severity (CES-D total score) as the outcome variable. We recorded parameters such as total effects, direct effects, and indirect effects in the model and calculated partially standardized indirect effects as well as the proportion mediated. Two-sided *p* < 0.05 was statistically significant.

## Results

### Demographic and clinical characteristics

A total of 300 children and adolescent depression patients were consecutively recruited in outpatient clinics and wards of various centers. Of these, 285 participants completed all questionnaires, and 15 patients who responded to the scale of bad or non-compliance were rejected. Table 1 shows about 78.9% of patients with depression combined with childhood trauma. Compared with patients without childhood trauma, the age of patients with childhood trauma was lower (*Z* = −2.04, *P* = 0.042). The incidence of childhood trauma was significantly higher in patients with parents who were divorced/ remarried, separated, or otherwise (*X*^2^ = 7.44, *P* = 0.045). In addition, compared with those without childhood trauma, depression patients with childhood trauma had significant differences in sleep disorders, insomnia, insomnia total score, and CES-D total score (all *P* < 0.001; *X*^2^ = 17.59, *P* < 0.001; *X*^2^ = 14.45, *P* < 0.001; *Z* = −3.97, *P* < 0.001; *Z* = −5.83, *P* < 0.001).

**Table 1:** Demographic and clinical characteristics in the total sample.

	**Total sample (*N* = 285)**	**Childhood trauma**		***Z/X^2^***	***P***
		**Yes (*N* = 225)**	**No (*N* = 60)**		
Gender *n* (%)				0.07	0.796
Male	77 (27.0)	60 (77.9)	17 (22.1)		
Female	208 (73.0)	165 (79.3)	43 (20.7)		
Age, mean (S.D.)	15.23 (1.71)	15.13 (1.71)	15.62 (1.67)	−2.04	**0.042**
Siblings *n* (%)				1.41	0.235
Yes	171 (60.0)	139 (81.3)	32 (18.7)		
No	114 (40.0)	86 (75.4)	28 (24.6)		
BMI, mean (S.D.)	20.09 (4.07)	21.05 (4.07)	21.22 (4.09)	−0.15	0.879
Physical disease *n* (%)				0.00	1.000
Yes	14 (4.9)	11 (78.6)	3 (21.4)		
No	271 (95.1)	214 (79.0)	57 (21.0)		
Grade *n* (%)				4.17	0.124
Middle school	129 (43.5)	108 (83.7)	21 (16.3)		
High school	133 (46.7)	98 (73.7)	35 (26.3)		
High school above	23 (8.1)	19 (82.6)	4 (17.4)		
Parental marriage *n* (%)				7.44	**0.045**
Married	212 (74.4)	159 (75.0)	53 (25.0)		
Divorce/remarriage	56 (19.7)	50 (89.3)	6 (10.7)		
Separation	7 (2.5)	7 (100.0)	0 (0.0)		
Others	10 (3.5)	9 (90.0)	1 (10)		
Sleep problem *n* (%)				17.59	**< 0.001**
Yes	240 (88.1)	200 (83.3)	40 (16.7)		
No	45 (11.9)	25 (55.6)	20 (44.4)		
Insomnia *n* (%)				14.45	**< 0.001**
Yes	215 (75.4)	181 (84.2)	34 (15.8)		
No	70 (24.6)	44 (62.9)	26 (37.1)		
ISI total score, mean (S.D.)	12.76 (6.00)	13.52 (5.86)	9.92 (5.67)	−3.97	**< 0.001**
Hypersomnia *n* (%)				2.77	0.096
Yes	117 (41.1)	98 (83.8)	19 (16.2)		
No	168 (58.9)	127 (75.6)	41 (24.4)		
ESS total score, mean (S.D.)	9.82 (4.66)	9.87 (4.83)	9.63 (4.00)	−0.48	0.632
CES-D total score, mean (S.D.)	36.48 (13.45)	39.01 (12.161)	26.98 (13.90)	−5.83	**< 0.001**

Bolded P < 0.05; BMI, body mass index; ISI, Insomnia severity index; ESS, Epworth sleepiness scale for children; CES-D, the center for epidemiologic studies depression.

### Relationship between childhood trauma and different sleep disorders

In Table 2, compared with patients without sleep disorders and insomnia, those with sleep disorders and insomnia had significantly higher scores on childhood trauma total score, physical abuse, emotional abuse, sexual abuse, physical neglect, and emotional neglect (all *P* < 0.05), while with or without hypersomnia had no significant effect on the total childhood trauma score and the subscale scores in patients with depression (all *P* > 0.05).

**Table 2:** Relationship between childhood trauma subtypes and different sleep disorders (Mann–Whitney *U*-test).

**Types**	**Sleep disorders**			**Insomnia**			**Hypersomnia**		
	**Yes**	**No**		**Yes**	**No**		**Yes**	**No**	
	**(*N* = 240)**	**(*N* = 45)**		**(*N* = 215)**	**(*N* = 70)**		**(*N* = 117)**	**(*N* = 168)**	
Childhood trauma total score	59.60	51.13	*U* = 3,142.50 *Z* = −4.451 ***P*** **<** **0.001**	60.31	51.97	*U* = 4,427.00 *Z* = −5.18 ***P*** **<** **0.001**	59.74	57.23	*U* = 8,576.00 *Z* = −1.83 *P* = 0.067
Physical abuse	7.55	6.56	*U* = 3,901.00 *Z* = −3.068 ***P*** **=** **0.002**	7.68	6.50	*U* = 5,214.00 *Z* = −4.01 ***P*** **<** **0.001**	7.44	7.36	*U* = 9,647.00 *Z* = −0.28 *P* = 0.784
Emotional abuse	11.67	8.80	*U* = 3,401.50 *Z* = −3.949 ***P*** **<** **0.001**	11.94	8.99	*U* = 4,675.50 *Z* = −4.77 ***P*** **<** **0.001**	11.68	10.89	*U* = 8,815.00 *Z* = 1.48 *P* = 0.138
Sexual abuse	5.91	5.40	*U* = 4,375.00 *Z* = −2.600 ***P*** **=** **0.009**	5.90	5.61	*U* = 6,437.50 *Z* = −2.34 ***P*** **=** **0.019**	5.95	5.75	*U* = 8,878.00 *Z* = −1.79 *P* = 0.074
Physical neglect	10.99	8.71	*U* = 3,178.50 *Z* = −4.398 ***P*** **<** **0.001**	11.20	8.89	*U* = 4,425.00 *Z* = −5.12 ***P*** **<** **0.001**	11.03	10.36	*U* = 8,644.50 *Z* = −1.74 *P* = 0.082
Emotional neglect	15.94	13.27	*U* = 3,877.00 *Z* = −3.008 ***P*** **=** **0.003**	16.10	13.71	*U* = 5,483.50 *Z* = −3.42 ***P*** **=** **0.001**	15.99	15.18	*U* = 9,051.50 *Z* = −1.14 *P* = 0.256

Bolded P < 0.05.

### Testing theoretical models: Sleep disorders as the mediator between childhood trauma and depression severity

#### Correlations between insomnia, hypersomnia, childhood trauma, and depression severity

Childhood trauma total score, insomnia total score, and depression severity (CES-D total score) were significantly correlated (Table 3; *r* = 0.479, *P* < 0.001; *r*= 0.577, *P* < 0.001), except hypersomnia (*r* = 0.081, *P* = 0.175). Therefore, in analyzing the relationship between childhood trauma and depression severity, insomnia as a continuous variable was selected as a mediating variable for further mediation analysis.

**Table 3:** Relationships between sleep disorders, childhood trauma, and depression severity of depression (Spearman's correlation analysis).

	**Childhood trauma total score**	**Insomnia total score**	**Hypersomnia total score**
CES-D total score	*r* = 0.479 ***P*** **<** **0.001**	*r* = 0.577 ***P*** **<** **0.001**	*r* = 0.081 *P* = 0.175

Bolded P < 0.05; CES-D, The center for epidemiologic studies depression.

#### Insomnia total score plays a mediating role between childhood trauma and depression severity

**Figure 1:**
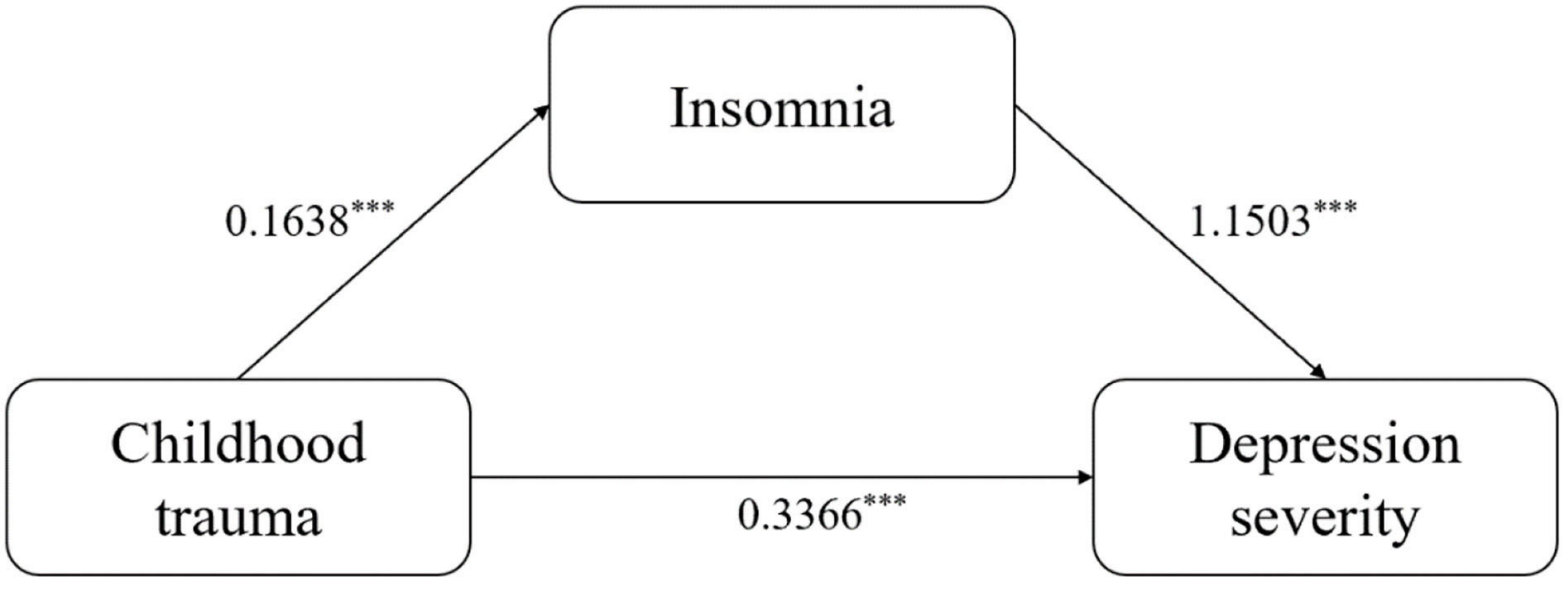
Mediation model results. ^***^: P<0.001.

Since the confidence interval did not contain zero [Table 4; 95% CI (0.1200–0.2606)], the total insomnia score had a significant indirect effect on the relationship between childhood trauma and depression severity, with insomnia playing a significant role in the mediating effect accounting for 35.90% of the effect of childhood trauma on depression severity. The results of the mediation model are shown in Figure 1. Bivariate correlations among covariates (gender, age, siblings, physical illness, grade, parental marriage, and family relationship) were assessed by Spearman's correlation analysis, rank sum test, and one-way ANOVA, not shown in the graph displayed in. We found significant differences in childhood trauma total score among different family relationships (*F* = 55.747, *p* < 0.001), so we re-included the family relationship as a covariable in the mediation analysis. However, after adjusting the covariance, the indirect effect of childhood trauma score on depression severity through insomnia was still statistically significant.

**Table 4:** Insomnia as a mediator of the relationship between childhood trauma and depression severity (total, direct, and indirect effects).

	**Total effect, estimate (S.E.), *p*-value**	**Direct effect, estimate (S.E.), *p*-value**	**Indirect effect, bootstrap confidence interval**	**Partially standardized indirect effect**	**Proportion mediated %**
Depression severity (CES-D total score)	0.5251 (0.0599), ***P*** **<** **0.001**	0.3366 (0.0531), ***P*** **<** **0.001**	0.1188–0.2599	0.0140	35.90%

Bolded P < 0.05; CES-D, The center for epidemiologic studies depression.

## Discussion

Our study is the first to explore whether sleep disorders mediate the relationship between childhood trauma and depression severity in children and adolescents with depression. In this study, we found a high incidence of childhood trauma in children and adolescents with depression; patients with childhood trauma are more likely to develop sleep disorders than those without. Sleep disorders are mainly characterized by insomnia rather than hypersomnia. Different types of childhood trauma can easily lead to insomnia. Insomnia is an important mediator between childhood trauma and depression severity.

Our study found that the incidence of childhood trauma in children and adolescents with depression is about 78.9%, which is consistent with previous studies (51, 52). The patients with childhood trauma are younger, indicating that younger age groups are more likely to suffer from childhood trauma. Traumatic events are associated with many short- and long-term negative consequences, including an increased risk of psychological and physical health problems, such as Internet addiction, self-harm, and other behaviors. At the same time, adolescent coping styles are immature and more likely to develop into these psychological problems (53–56). Therefore, psychological intervention should be carried out in time for young patients with depression to avoid more serious downstream results. In addition, our study also found that patients with poor parents' marriages are more likely to develop childhood trauma, which is consistent with the previous conclusion (57). Previous research has found that early childhood adversity is associated with depression scores, and different types of childhood trauma will increase depression scores (22). Our results further support the view that patients with childhood trauma have more severe depressive symptoms.

Patients with childhood trauma have a higher incidence of sleep disorders, mainly insomnia. The difference in this association may depend on the biological basis of insomnia and hypersomnia. Insomnia is characterized by a heightened level of physical, emotional, and cognitive arousal, consistent with theoretical consequences of trauma exposure (58, 59). In contrast, hypersomnia, usually manifested as a subjective feeling of lethargy from the inability to stay awake or to maintain attention, is associated with a decrease in neuronal activity that inhibits sleep-induced transmission and promotes wakefulness, which is unlikely to be the result of a trauma-related stress response (60). Therefore, childhood trauma and insomnia are more likely to share a common underlying mechanism. In addition, our study found that patients with depression who suffered from different types of childhood trauma were all prone to insomnia. Evaluating the relationship between specific subtypes of childhood trauma and adolescent sleep in young individuals with a history of depression found that emotional neglect is related to insomnia and can predict insomnia (61). Studies have shown that different trauma subtypes have different effects on biological stress responses and that they may interact in different ways (62). On this occasion, different trauma subtypes may affect sleep disorders differently.

Our study found that insomnia is partially mediated between childhood trauma and depression severity, which supports the theoretical model we created. These findings have implications for how trauma exposure affects depression severity and may suggest that post-traumatic sleep disorders in childhood interact with stress-regulatory systems to exacerbate depressive symptoms further. Studies on sleep responsiveness have found that sleep responsiveness refers to the degree of stress exposure that disrupts sleep and leads to difficulty falling asleep and sleep maintenance disorders. High sleep responsiveness usually shows a severe insomnia phenotype (63). Environmental stress affects the response of the sleep system to stress. People with high sleep response systems experience severe sleep deterioration under stress. At the same time, hypersleep responsiveness is associated with depression risk, and insomniacs show exaggerated neurobiological and cognitive-emotional responses to stress (64, 65). The neurobiological basis involved in this process includes the destruction of the cortical network, the imbalance of the autonomic nervous system, and the hypothalamic–pituitary–adrenal axis. Recent evidence on the relationship between the activity of the stress response system and the circadian rhythm system supports this reasoning (66, 67). The development of stress exposure and disease may fundamentally and functionally change the way the brain and body regulate stress and sleep. Based on the above, our findings suggest that insomnia may be one of the causes of the deterioration of depressive symptoms in children and adolescents suffering from childhood trauma.

There are some limitations to this study. First, this is a cross-sectional survey that cannot be used to assess causality; future longitudinal studies are needed to explore deeper. Second, the retrospective childhood trauma scale may have a recall bias, affecting the results. In addition, we use the self-reported scale to measure insomnia and hypersomnia, which is subjective and can be further studied more objectively in future. Finally, our study did not consider the effects of drugs, which may affect the results by improving patients' sleep disorders and depressive symptoms.

## Conclusion

The study found that the rate of childhood trauma and sleep disorders in children and adolescents with depression was as high as 70.2%. This study provides evidence for a simple mediation model suggesting that insomnia partially mediates the link between childhood trauma and depression severity. Effective identification and targeted treatment of insomnia symptoms in adolescent depressive disorder patients with childhood trauma can reduce the severity of depression and improve depressive symptoms. In future, longitudinal studies are needed to use more objective measurements of sleep disorders to clarify the relationship between childhood trauma, sleep disorders, and depression severity.

## Data availability statement

The data used for this study are available from the corresponding author on reasonable request. Requests to access these datasets should be directed to HL, huanzhongliu@ahmu.edu.cn.

## Ethics statement

The studies involving human participants were reviewed and approved by the Medical Ethics Committee of Chaohu Hospital of Anhui Medical University, and the Ethics number is 202009-kyxm-04. All subjects participated in the study voluntarily, and the informed consent was signed by the subjects or their guardians.

## Author contributions

HL: study design. BL, YY, DZ, QZ, ZhicL, SW, YS, and LX: collection, analyses, and interpretation of data. BL, YY, and DZ: drafting of the manuscript. KZ and HL: critical revision of the manuscript. All authors approved the final version for publication.

## Funding

This study was supported by the National Clinical Key Specialty Project Foundation (CN).

## Conflict of interest

The authors declare that the research was conducted in the absence of any commercial or financial relationships that could be construed as a potential conflict of interest.

## Publisher's note

All claims expressed in this article are solely those of the authors and do not necessarily represent those of their affiliated organizations, or those of the publisher, the editors and the reviewers. Any product that may be evaluated in this article, or claim that may be made by its manufacturer, is not guaranteed or endorsed by the publisher.
